# A Descriptive Model of Patient Readiness, Motivators, and Hepatitis C Treatment Uptake among Australian Prisoners

**DOI:** 10.1371/journal.pone.0087564

**Published:** 2014-02-27

**Authors:** Lorraine Yap, Susan Carruthers, Sandra Thompson, Wendy Cheng, Jocelyn Jones, Paul Simpson, Alun Richards, Hla-Hla Thein, Paul Haber, Andrew Lloyd, Tony Butler

**Affiliations:** 1 Justice Health Research Program, The Kirby Institute, University of New South Wales, Sydney, New South Wales, Australia; 2 National Drug Research Institute, Curtin University, Perth, Western Australia, Australia; 3 Combined Universities of Rural Health, Geraldton, Western Australia, Australia; 4 Royal Perth Hospital, Perth, Western Australia, Australia; 5 Offender Health Services, Queensland Health, Queensland, Australia; 6 Dalla Lana School of Public Health, University of Toronto, Toronto, Ontario, Canada; 7 Sydney Medical School, University of Sydney, Sydney, New South Wales, Australia; 8 Inflammation and Infection Research Centre, University of New South Wales, Sydney, New South Wales, Australia; UNC Project-China, China

## Abstract

**Background:**

Hepatitis C virus infection (HCV) has a significant global health burden with an estimated 2%–3% of the world's population infected, and more than 350,000 dying annually from HCV-related conditions including liver failure and liver cancer. Prisons potentially offer a relatively stable environment in which to commence treatment as they usually provide good access to health care providers, and are organised around routine and structure. Uptake of treatment of HCV, however, remains low in the community and in prisons. In this study, we explored factors affecting treatment uptake inside prisons and hypothesised that prisoners have unique issues influencing HCV treatment uptake as a consequence of their incarceration which are not experienced in other populations.

**Method and Findings:**

We undertook a qualitative study exploring prisoners' accounts of why they refused, deferred, delayed or discontinued HCV treatment in prison. Between 2010 and 2013, 116 Australian inmates were interviewed from prisons in New South Wales, Queensland, and Western Australia. Prisoners experienced many factors similar to those which influence treatment uptake of those living with HCV infection in the community. Incarceration, however, provides different circumstances of how these factors are experienced which need to be better understood if the number of prisoners receiving treatment is to be increased. We developed a descriptive model of patient readiness and motivators for HCV treatment inside prisons and discussed how we can improve treatment uptake among prisoners.

**Conclusion:**

This study identified a broad and unique range of challenges to treatment of HCV in prison. Some of these are likely to be diminished by improving treatment options and improved models of health care delivery. Other barriers relate to inmate understanding of their illness and stigmatisation by other inmates and custodial staff and generally appear less amenable to change although there is potential for peer-based education to address lack of knowledge and stigma.

## Introduction

Hepatitis C virus infection (HCV) has a significant global health burden with an estimated 2%–3% of the world's population infected, and more than 350,000 dying annually from HCV-related conditions including liver failure and liver cancer [1]. Prisons potentially offer a relatively stable environment in which to commence treatment, organised around routine and structure, and they usually provide good access to health care providers. The potential exists to maximise compliance to HCV treatment regimens [2]. It has been suggested that a new strategy for HCV prevention, ‘Test and Treat’, should be implemented whereby everyone within a certain age group in the high HCV prevalence category is tested and linked to treatment if they are infected [3]. This approach could substantially reduce costs in the long term. In addition, increasing HCV treatment within the prison setting could potentially act as a prevention strategy by reducing the prevalence of HCV in the injecting drug user population [4].

In Australia, HCV is the sixth most commonly notified communicable disease with 226,700 people estimated to be living with chronic HCV and is the primary cause of liver disease [5], [6]. Recent studies indicate that one in five men in prison in Australia (21%) and one third of women (34%) are HCV antibody positive [7]. Uptake of treatment of HCV remains low in the community and also in the prison [7]–[9]. A national survey of prisoners reported that only 5% of HCV positive prisoners had been treated for HCV [7]. Almost half of all Australian prison inmates report injecting drug use with around 70% incarcerated for drug-related crimes [10]. Given this nexus, HCV infection is very common among prisoners with an overall prevalence of 30%, and up to 80% among people who inject drugs (PWID) [11].

Standard care for the treatment of chronic HCV in Queensland (Qld), New South Wales (NSW) and Western Australia (WA) correctional centres during this study period was pegylated interferon (PEG-IFN) and ribavirin (RBV). The length of treatment was dependent on the HCV genotype: 48 weeks for HCV genotypes 1, 4, 5, and 6 with expected sustained virologic response (SVR) rates of 40%–50%; or 24 weeks (HCV genotype 2 and 3) and 80% SVR response rates [12]. This treatment regime was the same as that available to the general population who met the treatment criteria. Treatment could be deferred or delayed if the patient exhibited contraindications such as: decompensated liver disease, medical comorbidities which required medical attention (e.g. cardiovascular disease, diabetes, epilepsy, uncontrolled depression), non-adherence to medical visits, or active drug use [13]–[18].

A review of the literature highlights ‘readiness’ as an important construct in an individual's decision-making to undergo treatment but there is little consensus on its definition [19]–[21]. In one study, people with addictive behaviours were found to move through the following stages of readiness and change: from *pre-contemplation* (no intention to change) → *contemplation* (thinking of change) → *preparation for action* (behaviour, experiences or environment) → *maintenance* (prevent relapse and consolidate gains) [22]. Holt et al. (2007) in a comprehensive review stated that: *“Readiness occurs when the environment, structure, and organizational members' attitudes are such that…[patients]…are receptive to a forthcoming change” (p.290)* [23].

Models on barriers and facilitators to patient readiness and HCV treatment uptake are mostly based on studies of people who inject drugs (PWID) and other HCV infected individuals in the community. Anderson (1995), for instance, focused on predisposing and enabling patient characteristics (socio-demographics, socioeconomics, substance use, mental health, structural, unstable lifestyle) and perceived barriers and need (health beliefs, competing priorities, personal resources, perceived need, provider evaluation) [24]. Others included treatment eligibility (modifiable and non-modifiable treatment contraindications, liver disease, genotype, comorbid conditions) and environmental barriers (health insurance, physician access, transportation) [16].

In contrast, prisoners face unique challenges arising from the prison setting that influence HCV treatment uptake and which do not appear in any of the above models. Multiple barriers to treatment uptake in prison have been reported including: treatment refused due to short prison stays; the prison bureaucracy delaying treatment; and prisoners refusing transfers to other correctional facilities for treatment [25]–[28]. Enablers to treatment included prisoners relinquishing their parole eligibility to commence treatment inside prison [27]. Nevertheless, in common with those infected in the community at large, prisoners also experience poor access to treatment inside because of stigma, low screening uptake rates, fears of the disease, and a lack of awareness and knowledge about HCV infection and medical procedures [26].

Between 2010 and 2013, as part of the Hepatitis C, Prisons, and Treatment Opportunities Study (HePATO), we interviewed inmates diagnosed with HCV in Qld, NSW, and WA and explored issues associated with HCV treatment uptake including: awareness of HCV treatment services in prison, reasons for deferring, delaying, refusing and discontinuing treatment, and motivating factors to undergo treatment in prison. We triangulated the data with perspectives from health service providers responsible for delivering HCV treatment to prisoners.

## Methods

Between 2010 and 2013, 116 inmates were interviewed from prisons in NSW, Qld, and WA (Table 1). In addition, 29 health professionals were interviewed in the three states who were directly involved in HCV diagnosis and treatment programs inside prisons, and six outside health professionals who were either associated or involved in the education, care or follow up of prisoners with HCV outside and within the prison system (Table 2).

**Table 1:** Socio-demographic profile of prisoners interviewed in New South Wales, Queensland and Western Australia for the HePATO Study.

Characteristics	NSW		Qld		WA		TOTAL	
	Men	Women	Men	Women	Men	Women	Men	Women
**Gender**	37	15	31	9	21	3	***89***	***27***
**Age Group**								
21–30	12	1	9	5	6	3	***27***	***9***
31–40	17	6	8	2	8	-	***33***	***8***
>40	8	8	14	2	7		***29***	***10***
**Indigenous status (Yes)**	11	6	11	4	1	2	***23***	***12***
**Education**								
Year 10 and below	30	9	27	6	15	2	***72***	***17***
Year 12 (HSC/TEE/TEA etc.)	1	3	2	-	3	-	***6***	***3***
Trade/professional qualification	4	2	2	1	3	1	***9***	***4***
University level	2	1	-	1	-	-	***2***	***2***
Refused	-	-	-	1	-	-	***-***	***1***
**No. of times in an adult prison**								
Once	4	5	8	1	3	2	***15***	***8***
2 or more times	33	10	23	7	17	1	***73***	***18***
Other/Refused	-	-	-	1	1	-	***1***	***1***
**Current sentence**								
<12 months	1	3	2	1	2	-	***5***	***4***
1–5 years	14	6	11	4	11	2	***36***	***12***
>5 years	8	2	11	1	8	0	***27***	***3***
Not yet sentenced	14	4	7	2	-	-	***21***	***6***
Other/Refused	-	-	-	1	-	1	***-***	***2***
**Treatment status in prison**								
Cleared naturally	1	-	3	2	-	-	***4***	***2***
Treatment not offered or unavailable	-	-	5	3	2	2	***7***	***5***
Deferred/Delayed treatment	7	1	3	-	-	-	***10***	***1***
On waiting list	9	5	7	2	2	-	***18***	***7***
On treatment/Completed/Discontinued	12	6	9	-	15	1	***36***	***7***
Refused by medical staff	2	1	-	-	-	-	***2***	***1***
Refused by prisoner (self)	6	2	4	2	1	-	***11***	***4***
Other/Refused	-	-	-	-	1	-	***1***	***-***

**Table 2:** Prison health staff and other professionals interviewed in NSW, QLD and WA for the HePATO Study.

Interviews	NSW	QLD	WA	*Total*
**Prison health professionals**				
Physician	-	1	2	***3***
Nurse Unit Managers/Nursing Staff (Population, Mental Health, Hepatitis C, Primary Health Care/Enrolled Nurses)/Clinical Nurse Consultants	15	5	5	***25***
Drug and Alcohol Counsellor	1	-	-	***1***
Offender health services manager	-	1	-	***1***
**Outside health professionals**				
Justice Health Connections	2	-	-	***2***
Community hepatitis C nurse	-	1	-	***1***
Hepatitis Council	-	3	-	***3***

### Recruitment

To obtain an adequate cross-section of respondents, the interviewers (LY & SC), prior to their visit, provided information to prison nurses indicating the types of participants they wanted to interview including gender, age group, Indigenous background and HCV diagnosis and treatment status, as per the study protocol. Specific exclusion criteria included inadequate proficiency in English, inmates with severe mental health issues, and inmates who on the advice of custodial staff may endanger the safety of the interviewer. Initial recruitment was conducted through the prison nurses who referred participants to researchers. The most common reason for inmates not participating was prison work or other activities taking priority. Some respondents were called to the clinic but did not attend, even after agreeing to be interviewed beforehand – the reasons for non-attendance were not sought. The demographic characteristics of participants are listed in Table 1.

On the day of the interview, inmates were contacted by a prison nurse and asked to visit the prison clinic. The study was explained in detail, and if agreeable, written consent was provided. Interviews were digitally recorded in NSW and Qld, but handwritten notes were taken in WA since digital recorders were not permitted inside the prisons. Inmates who did not want to participate or wanted to stop the interview at any time could do so without penalty, they were not disadvantaged in their health care or other services inside prison in any way. No remuneration was given for participation. Interviews were conducted in private clinic rooms or offices located within the prison accommodation area. No custodial or clinic staff were present during the interviews, only the prisoner and researcher. Video surveillance equipment without sound enabled monitoring of the interviews to ensure the safety of the researcher.

Prisons in NSW, Qld and WA varied in terms of the availability of both HCV treatment and methadone treatment (Table 3). Of the 16 prisons visited, two did not offer HCV treatment and five prisons did not offer methadone treatment. Two different HCV treatment models of care were observed: (a) *a medical model of care* where treatment was initiated by prisoners visiting community-based specialists, followed up and treated by a nurse and other health providers in the prison system; and (b) *a nurse-led model of care* (NLMC) whereby prisoners were assessed and managed largely by skilled nurses in the prison system with more limited specialist involvement (Table 3) and is described in more detail elsewhere [29].

**Table 3:** Prisons visited as part of the HePATO Study.

Description	NSW		Qld		WA		TOTAL	
	Men	Women	Men	Women	Men	Women	Men	Women
**No. of prisons visited**	**5**	**2**	**4**	**1**	**3**	**1**	***12***	***4***
No. of prisons with hepatitis C treatment	5	2	3	-	3	1	*11*	*3*
No. of prisons with methadone treatment	4	2	-	1	3	1	*7*	*4*
**Models of hepatitis C treatment care**								
No. of prisons Nurse Led Model of Care	4	2	1	-			*5*	*2*
No. of prisons Medical Model of Care (Prison to Hospital)	1	-	2	-	3	1	*6*	*1*
**Prison classifications**								
Minimum/Low security	1	-	1	-	1	-	*3*	
Medium	2	1					*2*	*1*
Maximum/High security/Maximum (Remand)/High security (Remand)	1	1	3	1	1	-	*5*	*2*
Mixed (May Include Remand)	1	-			1	1	*2*	*1*

### Data collection

Interview guidelines were developed and shared between the trained interviewers (LY & SC) covering: experiences of living with HCV; diagnosis and treatment experiences; perceived barriers to treatment in prison; and opportunities for treatment (see Table S1). The average time of the recorded interviews among prisoners was 44 minutes, ranging between 8 minutes and 2 hours. Health professionals were asked about their experiences with prisoners, what they perceived were the barriers to, and opportunities for provision of treatment in prison. A grounded theory approach informed the collection and analysis of qualitative data [30]. This involved reading a textual database and exploring and discovering categories, concepts and properties and their interrelationships within the data.

### Analysis

This qualitative analysis did not seek to obtain a representative view, but rather to gather rich and complex data via in-depth interviews in order to enrich the researchers' capacity to investigate and gain an understanding of respondents' feelings, opinions and feedback. Throughout the study, transcribed data was coded within QSR NVIVO 9.0. The thematic coding structure was revised as each interview was analysed. Analysis of data ceased when thematic saturation was reached.

### Human research ethics

Ethics approval was obtained from the Human Research Ethics Committees at Curtin University in Perth, Western Australia (HR201/2008), the Department of Corrective Services NSW (10/10958), Justice Health NSW (D4578/10), Metro North Health Service District in Queensland (HREC/10/QNRC/40), the Aboriginal Health and Medical Research Council Ethics Committee of NSW (744/10), and ratified by the Human Research Ethics Committee at the University of New South Wales, Sydney.

## Results

**Figure 1:**
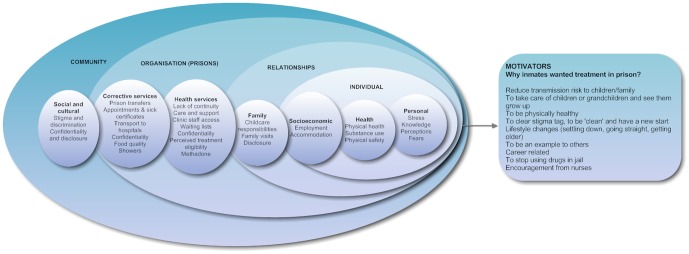
A model of prisoner perceptions on the barriers and motivators for taking up hepatitis C treatment inside prison.

A model of prisoners' perceptions which influenced their readiness to take up treatment in prison was developed to facilitate our understanding of the complex and interactive nature of prison HCV treatment uptake and its motivators (Figure 1). Multiple domains of influence were evident with overlapping interrelationships between the individual, their relationships, the organisation (corrective and health services) and the community. We discussed the model in relation to prisoners' perceptions which led them to delay, defer, refuse or discontinue treatment inside prison.

### Individual level (Personal)

#### Stress

Stress, particularly on entry to prison, was common among inmates. Stress symptoms included sleep disturbance, poor concentration, and preoccupation with ongoing problems (*AB, female, 33 years, Qld*). Prisoners were not interested in taking up treatment if stressed by issues deemed to be more important than HCV. As one mental health nurse explained:

*…a lot of them don't want to start treatment especially while they're on remand because of the stress of court… They want to be able to present well when they go to court. Also family stresses…they can be under a certain amount of stress and they don't want any additional stress. They want to be emotionally available if something's going wrong with the family.* (Nurse, NSW)

#### Knowledge

Lack of awareness of HCV treatment in prison was also a barrier among infected prisoners. Respondents knew their HCV diagnosis from prison medical staff on entry, but some had not had prison-based treatment fully explained. Only after a period of incarceration (which varied from weeks to months or even years) did they learn from other inmates, or a nurse that treatment was available. Others did not have sufficient information to decide on treatment or had been misinformed about treatment. One inmate was unsure if the treatment was safe or not *(RT, male, 31 years, NSW)*, while another was misinformed about the duration of treatment in prison *(BW, male, 57 years, Qld)*. Others were misinformed by their health care provider about HCV such as being told that the disease was incurable *(SN, male, 51 years, Qld*) or too hard to treat *(MA, male, 51 years, Qld)*.

In general, HCV treatment was not widely advertised inside prisons. In one prison, medical staff explained why they did not usually discuss HCV treatment on entry:

*…if you suddenly put treatment in a booklet and people are seeing this, they believe that that is their right to be given treatment […] And so you don't want to incriminate yourself because they will pick this up and say, “Why didn't I get treatment? It tells me here I have to have treatment.”… You're better off leaving it as an open slate and then you talk to them, and then you let them know that treatment is available if they meet the criteria.* (Nurse, Qld)

#### Perceptions of treatment

Individual understanding of HCV disease and the effects of treatment were factors amongst some prisoners refusing treatment. One inmate believed that he could control his infection if he led a healthier lifestyle *(CB, male, 51 years, NSW)*, while another thought that treatment would be detrimental to his health *(RM, male, 47 years, Qld)*. Another perception was that HCV treatment regimens appeared to be improving over time, with one inmate indicating that he was “*hangin' in there”* (for better treatment) *(FR, male, 37 years, NSW)*. Another participant reported deferring treatment as he saw little negative impact in being infected, he saw other infected people who appeared healthy *(KW, male, 25 years, NSW)*. While others had a *‘did not want to know’* attitude since it would add to their worries *(MQ, female, 31 years, NSW)*.

#### Treatment related fears

Prisoners furthermore reported fears associated with treatment. Inmates dreaded medical procedures such as liver biopsies, the size of the needles associated with interferon injections, or feared relapse to injecting drug use as a consequence of injecting during treatment. Although liver biopsies are no longer required for patients to qualify for treatment, one inmate had delayed the decision to undertake treatment as she was unsure of this *(ER, female, 35 years, NSW)*. The size of venepuncture needles and negative experiences with nurses trying to draw blood for laboratory testing led some inmates to defer, delay, or refuse treatment.

*Well they find it very hard to get blood from me. Very, very, very hard to take blood from me. They tried three times today and still couldn't get no blood, and… that was enough for me.* (WG, female, 35 years, NSW)

Other fears were related to inmates having to inject themselves during treatment leading to drug relapse. These fears were usually calmed by the prison nurse and patients subsequently underwent treatment *(Nurse, NSW)*.

Many reported fears of the adverse side effects of treatment, including nausea, weight loss, and difficulties in conceiving children after release. Prisoners recalled that these fears were typically exacerbated by seeing, or being told of, bad side effects experienced by others. Among men, weight loss was feared - one inmate was afraid other prisoners might assume he was HIV infected if he lost weight.

*The weight loss. [ …But you gain that weight back again.] I know but…you've got 300 blokes looking at you every day and…it's not that I really care what they think but there's another fella, … I've seen him a few times and … I've thought to myself, “He looks like he's doin' the interferon treatment,” …‘cause he looked bad, you know. It makes you look shockin’. People start thinking you've got AIDS or somethin', you know.* (JB, male, 40 years, NSW)

Losing weight was regarded as contributing to vulnerability and increasing the prospect of physical assault inside prison.

*…to keep yourself safe from assault and victimisation, you have to keep really fit and healthy… I've had people express that concern. One guy stopped the treatment ‘cause he was having an enormous amount of weight loss.* (Nurse, NSW)

Otherwise, prisoners did not want their families to see them looking sick and losing weight during prison visits, as they would be forced to reveal that they were infected and undergoing HCV treatment.

*…their families don't know they've got hep c and they don't want their family to know, so they don't want to have the treatment. One guy said to me…“I don't want to have the treatment ‘cause it's going to make me lose weight and look sick, and I don't want my family to see me on visits looking and feeling like that.* (Nurse, NSW)

Amongst other prisoners, they did not want treatment since they feared difficulty conceiving children with their partners after they were released *(FR, male, 37 years, NSW)*. A mental health nurse observed that women, in particular, deferred treatment to have children first.

*I've been told, there's some…dynamics… of some of the relationships can be, shall we say, interesting…. And, for some of the women, the way they seal a relationship is by having a child with their partner. So they want to have the child before they get treatment.* (Nurse, NSW)

### Individual level (Health)

#### Physical health

Some prisoners were not ready for treatment as they thought that their HCV was not of the severity at which treatment was necessary but would consider it if they experienced worsening physical health.

*[How come you're not as interested?] Probably because I feel healthy. I don't know. […but you said you wanted to do a liver function test.] Yeah, I just wanted to see if it was bad. Because if it is getting real bad then I'll see somebody about it, you know. Maybe get into treatment or whatever. [..] But it isn't really giving me any problems.* (PV, male, 37 years, Qld)

#### Substance use

Many prisoners stated that it was pointless getting on treatment if they were still using and sharing injecting equipment or if they were still drinking when out of prison - since these habits were detrimental to their liver and could lead to re-infection if injecting.

*No…I've never been on treatment, no. [Would you like to be on treatment?] Yeah, I would but … I don't want to go through something full-on like that if I'm just going to share with someone again, you know…[And you think you will be sharing with someone again?] Yeah, probably…I don't want to waste anyone else's time and myself.* (RT, male, 26 years, Qld)

#### Physical safety

Other prisoners refused to be transferred to another prison for treatment because of personal safety, refusing to leave their current location which they regarded as home and a place they felt safe.

*… because they're just such high profile classo* [classified] *patients that they may not come out of* [prison name] *alive or they have association problems. So the inmates* [in the treatment prison] *want them elsewhere. So they're here* [in this prison] *and they're protected. The other thing is that this is their familiar place – it's become their home…so we take them out of their home and move them to [treatment prison name], they don't cope well… Their coping skills are poor….So they see it* [current prison] *as their home. When they leave the jail, they've got to worry about who's going to come up behind them (in another prison).* (Nurse, NSW)

### Individual level (Socio-economic)

#### Employment and accommodation

Food and accommodation are provided to all prisoners by corrective services but prisoners can work to earn additional income which can be used to pay for luxuries (e.g. tobacco). One prisoner (now on treatment) had delayed her decision for fear of losing her job since treatment would require taking time off work and waiting in the clinic to consult a prison health care provider *(KJ, female, 34 years, NSW)*. Inmates were reluctant to transfer to another prison offering treatment since they risked losing their preferred accommodation and employment and having to work through the system again.

### Relationship level (Family)

#### Family and relationships

Other prisoners refused treatment because of family commitments on the outside. One refused a transfer to consult a hepatologist since he was concerned about his partner's health and difficulties they would have with transport to reach the (treatment) prison *(MH, male, 44 years, NSW)*. While a female inmate was concerned with having less energy to care for her young children on the outside after being released and after completing treatment in prison *(WG, female, 35 years, NSW)*.

### Organisation level (Health and Corrective Services)

#### Lack of continuity

Not all prisoners deferred, delayed or refused treatment in prison. Some prisoners were highly motivated and were proactive in seeking treatment inside prisons, but had experienced delays.

*Basically, I wanted to get on the program and get rid of the hep. […] [But why did you wait so long? I mean it's four years.] Oh I've asked before* [at the prison clinic]. *[You've asked before? And what happened then?] Nothin' much… When I first come up here I asked about it again, you know.… Just gets swept under the carpet. A couple of years later, finally got going with it. [Why do you think there was a change?] When someone dies I suppose people sit up and take notice.* (WJ, male, 52 years, Qld)

This lack of continuity might be due to the prison system itself. During the interviews, many prisoners did not keep their appointments at the prison clinic because of sudden prison lock downs, prisoners being away for other activities (court appearances, legal interviews, or drug education courses), or prison officers not informing the inmate they had been called. On the other hand, prisoners may have decided not to attend clinic appointments, as told by one female prisoner:

*… a couple of times too I didn't go when they called…I refused a couple of times, just couldn't be bothered to go…[But if they told you…?]…Yeah, if they'd told me I would have gone but a lot of the time they don't. They say, “You've gotta go to the clinic.” And I think it's either the psych* [psychiatrist] *or the physio (physiotherapist)… and I think, “No, I'm not going”…And [the prison nurse] gets the shits and they end up calling me back up and saying, “Get your arse up here.” Then I go. But I think it would have been a lot quicker* to get on treatment] *if I showed up to all the appointments.* (KJ, female, 34 years, NSW)

Another explanation may be that prisoners reported constantly being transferred between prisons, and hence missing planned follow-ups. These transfers were reported to be due to a multitude of reasons, such as bed availability in prisons, court or medical treatment in another town, prison re-classification, or as punishment for behavioural infringements.

#### Waiting times

Other prisoners were deterred from undertaking treatment because of the long waiting list *(TT, female, 39 years, NSW*), with one inmate reportedly having to wait two years before commencing treatment *(BW, male, 57 years, NSW)*. Prison health care nurses (Qld, NSW, WA) explained that waiting times were compounded by patient medical, mental or drug use comorbidities. Comorbidities had to be addressed before they could commence treatment or prisoners were placed on a community-based hospital waiting list which could sometimes be as long as two years. In NSW, sometimes nurses did not properly complete the ‘waiting list entry’ form which followed prisoners site to site and which might delay consultations with a nurse *(Nurse, NSW)*.

#### Hospital visits

In some centres gaining access to a specialist physician for assessment meant many prisoners had to visit an external hospital as there were no relevant specialists available at the prison. For some prisoners, the initial experience of visiting a hospital outside prison was a factor in their decision not to continue with the pre-treatment work ups:

*They wake you up at 4.00am in the morning here. Handcuff you…You wait ‘til the officers do their rounds. Then they put you in a holding cell ‘til about 6.30am… The truck comes about 6.30am, 7.00am. So you're up at 4.00am. So you've sat in the jail here for three hours before they move you…You get loaded onto the truck and they drop people who are going to court at Roma Street and…to the [hospital name]…(at)… 8.30am, 9.00am. Then you just go in there and you wait…I saw the doctor for 10 minutes…So I was away 14 and a ¼ hours for a 10 minute appointment…You get back about 6.00pm… it's…just a terrible, terrible day. Plenty of blokes, they refuse to go.* (ML, male, 52 years, Qld)

#### Staff access

Access to clinics varied between prisons. A nurse in NSW thought that women's prisons had better clinic staff access than men's prisons. One prisoner felt that prison officers and nurses in some prisons had become desensitised and fatigued by prisoners' demands over the years to the point that requests were sometimes ignored. This same prisoner had to wait until he was in another prison which had good clinic staff access before he pursued treatment.

*You won't get to the clinic in other gaols. It's really hard.…you'll get some blokes that are here twice a day. “I need a Panadol. I need something.”… All the time … They're constantly seeing the screws* [prison officers]. *Constantly seeing the sweepers* [cleaners]. *“I need, I need, I need …” That's all you hear from them.* [Nursing] *staff get pretty tired of hearing that all the time?… And then they abuse them and carry on.* (PW, male, 28 years, NSW)

#### Lack of care and support

Inmates believed that their relatives or partner on the outside could provide better care and support and they would have better food and showering facilities while on treatment, providing a level of comfort not found in prison while they experienced the worst of the treatment side effects *(FR, male, 37 years, NSW)*. No formal treatment support groups or networks for prisoners were reported to exist in prison but *ad hoc* support groups were formed among inmates who met while waiting in the clinic before treatment or elsewhere.

*… when she* [nurse] *told me…there was a chance that I could be sick* [from treatment] *and how long it was going to take, I wanted to leave* [treatment] *for when I got outside…so that I had a carer. […Who would… be your main carer then?] Well my family and my girlfriend. [You didn't want to do it inside?] That's the thing about inside… because it's such a long period of 12 months, you know… that's long…I've seen some of the inmates that have had treatment. They look pretty sick.* (TN, male, 30 years, NSW)

Not being able to stay in their cell if they were sick was an issue repeatedly raised by inmates in NSW who wanted treatment but had made the decision to wait until they were released.

*…I wouldn't take the risk of gettin' crook in gaol. Jail's bad enough without bein' sick in here. [But if you were crook outside, wouldn't that be worse?] No, it'd be better. [In what way?…]if you're crook* [inside prison], *you can't just stay in your room and sleep or whatever you wanna do, you know. Things like that. Unless you're near dyin'… jail's bad enough without feelin' sick, you know…Unless you're really sick then you've gotta come and see the nurse, you've gotta get a thing signed, and then they'll lock you in your room for the rest of the day… gaol's horrible and you don't wanna be sick while you're in gaol.* (AG, male, 57 years, NSW)

#### Treatment eligibility

Inmates reported that they had delayed or deferred treatment because of medical co-morbidities (cancer, anaemia), psychiatric co-morbidities (depression) or drug related co-morbidities (current injecting drug user). Furthermore, some were on remand or had short sentences, and therefore did not have enough time in prison to complete the treatment.

*I started it in* [prison name]. *I started all the preliminaries in* [prison name] *but I had to wait ‘til I got here before I could actually get onto the program…And even then I had to jump up and down, and scream. […How long did it take you to get on the program?] About 14 months….[…]If I'd have been able to kick off when I wanted to start it…I would have been clean at* [prison name] *and not had to worry about going through* [another 3 prisons] *before I could get on the program…They wouldn't let me…do the program while I was on remand. I had to wait to be a sentenced prisoner…And that's a bit of a barrier ‘cause at the start, you wanna do it, you wanna do it, but keep being told ‘no’ defeats you a little bit.* (DR, male, 38 years, NSW)

Even if an inmate was eligible for treatment according to standard treatment protocols, clinic staff may still not deem them to be an acceptable treatment candidate. Clinic staff were observed to initially screen HCV-infected prisoners based on their sentenced/non-sentenced status, length of incarceration, current injecting drug use, psychiatric comorbidities and other factors. Some nurses decided that some inmates did not have enough time before release to stabilise their addictions and psychiatric co-morbidities, complete their pre-treatment workups, wait for treatment (waiting list varied between several months and 2 years), and complete the 6- or 12-month course of combination therapy of pegylated interferon and ribavirin.

Clinic staff therefore screened prisoners whom they believed were more likely to complete treatment inside prison in order to maximise limited resources and to report more successful outcomes (i.e. completion of treatment). The option of inmates continuing treatment outside of prison once released was rarely discussed as staff believed that they frequently missed medical appointments outside to deal with more important priorities like employment, accommodation, drug use, and family. These were seen as usually getting in the way of their treatment after release *(Two nurses, NSW)*.

#### Methadone

Some inmates refused treatment themselves while others wanted to get on treatment but were refused by health providers until they were drug-use abstinent or on a methadone program to stabilise their addiction and injecting drug use. Methadone was available in all three Australian states studied except for men's prisons in Qld and one prison in NSW where methadone was banned by the prison management much to the frustration of inmates who wanted treatment:

*Bring the program* [methadone] *back in and it'll stop people using… ‘cause no-one's gonna get on it* [treatment] *while they're still usin’. You know what I mean?…What's the use?* (KW, male, 25 years, NSW)

Furthermore, methadone treatment policies in NSW meant inmates had difficulty accessing the methadone treatment program if they had not commenced such treatment in the community prior to coming into prison.

### Community level (Social and Cultural)

#### Stigma and discrimination

Willingness to go on treatment was also affected by the shame and stigma of being an injecting drug user, being infected with HCV, and being labelled ‘a junkie’, fostered among the prisoners themselves – even by those who had been or were current injecting drug users. Inmates therefore did not want to talk openly about HCV with those they did not trust and this led to uncertainty about having treatment in prison.

*For some, having hepatitis C means you are a ‘junkie’ so most keep the information private.* (JL, male, 42 years, WA)

Others were hesitant about treatment since they felt shame in being infected and were afraid of their family members' reaction if they found out.

*Some of them their families don't know that they've got hep c. Some of them are ashamed of having hep c and on how they've caught it too… their families don't know that they've been using drugs.* (Mental Health Nurse, NSW)

#### Confidentiality

The lack of confidentiality was felt keenly among patients who feel shame and stigma upon disclosure which had an impact on their willingness to undergo treatment. Prisoners usually lived together in close quarters and would see each other every day. It was evident, in our interviews, that prisoners were keenly observant of changes in another person's health and appearance, which could create suspicion, making it difficult for the patient to keep his or her HCV status confidential. As one prisoner on treatment pointed out,

*…the poor blokes that have gotta do it* [treatment] *for a year, like you start treatment and by the time you finish it everyone knows. You know what I mean? … it may not be big in your eyes but to the person that's actually doin' it, it's pretty degrading.* (SM, male, 33 years, NSW)

Lack of confidentiality can also be found among prison officers and nurses. For example, this same inmate was asked by prison officers what the medical issue was before being granted access to the nurses in clinics. This can deter some prisoners who may initially have wanted to seek treatment in prison.

*…they* [prison officers] *want a reason … So they know why you're coming in that door. I have to physically say* [to prison officers], *“I'm coming here to have my hep c shot. All right? You happy now?”* (SM, male, 33 years, NSW)

Whilst some nurses had a dismissive attitude believing that *‘everyone knows’* they are infected with HCV *(Nurse, NSW)*.

### Treatment motivators

Those on a waiting list, currently on treatment, or who had completed treatment indicated that they were motivated to take up treatment in prison, mainly due to concerns for the future. This future was related to: their family, partners, children and grandchildren; their health; release from prison; drug use cessation; career goals; and the impact of life changing events. Some were also motivated through the encouragement of the prison nurse to accept treatment.

#### Protecting family and children

Protecting partners, children and family from potential blood-borne viral transmission was repeatedly mentioned by inmates as their main reason for wanting treatment. They were afraid that they could transfer the virus to their partner and to their children, particularly during rough play.

*[And so this was last, this is just last week? So it's pretty sudden isn't it wanting treatment when previously you did not care)?] Yeah. [How do you feel about that?] Yeah, good. ‘Cause I got another kid coming on the 14th of March… [Why is that important?] Just so like if I have got cuts and whatever on me I'm not gonna expose myself to them and that sort of stuff. And catch it and that.* (MV, male, 29 years, Qld)

For older inmates, being treated for HCV was important too. One grandmother felt that with treatment she would be healthy enough to raise her grandchildren outside. Another inmate said he wanted to live longer and be able to see his grandchildren grow up.

*I need to do it actually… ‘cause I want to be around when my little grandkids grow up and they're playing football and my grand-daughter's playing basketball, and my grandson's boxing, you know. Yeah, I wanna see that, yeah.* (IT, male, 40 years, NSW)

#### Health and wellbeing

Even for prisoners without children or family, their motivation to go on treatment was to prevent HCV-related ill health and to prolong their life or to keep healthy. Other inmates who sought treatment did so as they were already experiencing ill health and symptoms from their infection (*PH, male 44 years, NSW; DC, male, 27 years, WA*).

#### Career related

Getting healthy might not be the only reason prisoners commence treatment. One inmate discussed his future as a personal trainer after he was released and described his perceived need to be rid of the disease so he could use steroids without damaging his liver *(NC, male, 27 years, NSW)*.

#### Life changing events, significant others and growing old

Another group of prisoners appeared to have experienced life changing events which served as motivators for taking up treatment in prison after many years of deferring treatment. One prisoner was motivated since he now had a partner and children which led to additional responsibilities. He was also influenced by a life shaping event to re-evaluate his lifestyle since he had lost his mother to cancer while in prison, which forced changes in his mental attitude towards his own health, lifestyle and criminal activities *(RT, male, 31 years, NSW)*. Growing old and ageing was another motivating factor to commence treatment *(SP, male, 44 years, WA)*.

#### Removing stigma, new start and being ‘clean’

Other inmates commenced treatment to rid themselves of the ‘junkie’ stigma and reminders of their former drug use *(AA, male, 28 years, WA)*. These individuals said they wanted to be ‘clean’, and this had physical and psychological consequences extending into the ‘right’ decisions they made into the future, particularly after release.

*[Is there a particular reason why you want to clear it?] I just don't want to have hep c. I don't want that tag. [Is there some sort of stigma?] Yeah, of course there's a stigma…I hear that you got hep c. “Oh no, you're a junkie.* (DR, male, 38 years, NSW)

Prisoners, particularly those who were about to be released, believed that being treated would lead to a new start after prison, and was part of their perception of being ‘clean’. Thus treatment was one of the things which needed to be done to help build a new future.

*What made me do it?… I was doin' long enough and I just had a reality check. I wanted a new start I guess and that was one thing I wanted out of me way.* (PH, male, 44 years, NSW)

*If I was never going to be released I would not get treated (prisoner now on treatment and soon to be released).* (GB, male, 33 years, WA)

#### Peer based knowledge

One Indigenous elder, in addition to wanting treatment to be healthy and to see his grandchildren grow up, mentioned that he wanted to help other Indigenous men in prison. After going through treatment, he felt he would be able to talk about his experiences to others and encourage them to go on treatment (*IT, male, 40 years, NSW*).

#### Stop using drugs in jail

The majority of prisoners interviewed did not want treatment if they were still using drugs as they were at risk of being reinfected. However, one inmate believed that being treated for HCV would encourage him to stop using drugs inside jail *(WS, male, 40 years, NSW)*.

#### Encouragement from nurses

In some prisons, nurses had established good relationships and trust with inmates particularly amongst those serving longer sentences and recidivist inmates who consulted the same nurse for each incarceration episode. The encouragement of a prison nurse was frequently described as a factor among inmates and their acceptance to undergo treatment.

*[…what took you over the line like from thinking about it to actually doing it?] Oh, (the prison nurse was) always hassling me. Every time I went in the clinic, she's like, “K., come on, we've got to get you in here and we're going to do bloods.”* (KJ, female, 34 years, NSW)

## Discussion

This qualitative study, exemplified in the descriptive model (Figure 1), provides insights into prisoners' readiness and experiences with HCV treatment in prison, as well as, their decision to defer, delay, refuse or discontinue treatment. The model also describes factors which motivated change and seeking HCV treatment inside prison. Prisoners experienced many factors similar to those which influence treatment uptake of those living with HCV infection in the community [13], [15], [17], [23], [31]–[33]. Incarceration, however, provides different circumstances of how these factors are experienced which need to be better understood if the number of prisoners receiving treatment is to be increased.

The model describes how prisoners may not have necessarily experienced difficulties in obtaining the necessities of daily living whilst on treatment, but they nevertheless experienced unemployment, loss of income, and changes in accommodation – all of which influenced treatment uptake, albeit under different circumstances to those relevant in the outside community. Ironically, in an environment which is almost child-free, outside childcare commitments and responsibilities impacted on the willingness of women prisoners to accept treatment should they be released early.

Prisoners' attitudes, knowledge, perceptions and fears, and their physical health and mental outlook influenced treatment uptake. They lacked knowledge about HCV treatment and its availability inside prison and did not have enough information about treatment. They also had similar perceptions that treatment might be detrimental to their health, and believed that they could self-manage their disease by maintaining a healthier lifestyle. Many types of fear led them to defer, delay, refuse or discontinue treatment including, the fear of finding out if they were infected with HCV, fear of drug relapse, fear of various types of medical procedures, and fears related to the adverse side effects of treatment such as feeling sick in prison, weight loss, and difficulty conceiving children with their partner after release.

Like people infected with HCV in the community [31], [34], prisoners felt the shame and the stigma of being an injecting drug user and being infected. They feared being labelled a ‘junkie’ by inmates, being ‘unclean’, and were afraid of disapproval from family members if it was discovered they are or were a drug user and infected with HCV. This finding was unexpected given the large numbers of injecting drugs users in prison and the high rates of HCV. The stigma of being infected with HCV can lead to reluctance to talk about the infection among prisoners, compounded by the lack of confidentiality among prisoners, health, and corrective service staff which may dissuade inmates from taking up treatment inside prison.

Issues with health service delivery inside prison were similar to those reported by individuals in the community [14], [16], [17], [31], [34]–[37]. Prisoners experienced difficulties including, the lack of availability of HCV treatment in some prisons, lack of continuity by clinic staff, long waiting lists, the challenge of hospital visits for specialist consultations, difficulties with clinic access, concerns with the quality of care and support inside prison, and failure to maintain confidentiality.

Issues unique to prisoners include those who reported not being eligible for treatment by health care staff if they were on remand or had sentences considered too short for the workup and time to complete treatment. Furthermore, due to the prison environment, they were subject to involuntary prison transfers, faced transportation challenges from prison to community-based hospitals, and were subject to healthcare inequities such as, access to a methadone or other opiate substitution treatment which was available in the community but denied to prisoners in certain jurisdictions due to corrective services policies or directives. Another issue for prisoners was the myriad of stresses experienced during the different stages of their incarceration. Prisoners appeared to experience acute distress during the initial stages of imprisonment which served to reduce the priority they placed on health matters such as HCV treatment. For longer term inmates, these stresses may continue for years depending on their phase of incarceration.

Despite the many challenges posed by prison, many were motivated to take up treatment mainly out of concerns for the future. They took up treatment to prevent blood viral transmission to their family and children after release, to live long enough to see their children or grandchildren grow up, to become healthier, to ‘start’ a new life after release, as part of their career goals or a different outlook due to life changing events. The encouragement of the prison nurses was instrumental in inmates accepting treatment.

It was evident that the prison environment exerted a significant impact on health services and the lives of prisoners, which overwhelmed any differences in gender, education, class, and ethnic or Indigenous background which may have influenced treatment uptake in prison. Prisoners were all subjected to prison routines and regulations which affected their treatment uptake in varying degrees. Nevertheless, recommendations can be made to improve treatment uptake inside prisons.

Better health and custodial collaboration and communications may improve access to clinics and treatment, reduce the amount of prison transfers, and improve confidentiality among staff. Nurse led models of care such as that being implemented in NSW may overcome barriers by reducing prison to community hospital visits, improving the feelings of safety and comfort for some prisoners, and maintaining family visits for prisoners who wish to remain in the same prison and location.

In 2013, after the study data collection period, new therapeutic agents, direct acting antivirals drugs (DAAs), were approved for use in Australia for all genotypes and will be subsidised by the Australian government Highly Specialised Drug (HSD). The newly approved DAAs, used in combination with pegylated interferon and ribavirin treatment are associated with better treatment efficacy [38], [39] and may increase the willingness of inmates to take up treatment in Australian prisons. In the next 2–5 years oral (once-daily), tolerable, short-duration (12–16 weeks) DAA treatments with extremely high efficacy (cure >90%) and improved tolerability are likely to be approved for use in Australia [40]. This is likely to address some of the concerns expressed by prisoners in this study relating to side-effects.

Methadone should also be available to prisoners so that people who inject drugs can stabilise or cease their injecting drug use, reducing the potential for re-infection during and after treatment, and improving prisoners' eligibility for the treatment programs. With better education and promotion, it is possible to increase the knowledge inmates have on the availability of HCV treatment in prison, leveraging off their desire for a better future for their family, children and themselves.

## Conclusion

This study identified a broad range of unique challenges to treatment of HCV in prison. Some of these are likely to be diminished by improving treatment options and improved models of health care delivery. Problems with access to other relevant health care (including but not limited to opioid maintenance treatment and mental health care) and access to prison clinics generally will require broad scale improvement to the health care system inside prisons. Other barriers generally appear less amenable to change and are related to inmates' understanding of their illness and stigmatisation by other inmates, custodial staff and the community. Peer education and staff training, however, may be able to address these challenges.

## Supporting Information

Table S1 **HePATO Qualitative Research Interview Question Guidelines.**
(DOCX)

## References

[1] Averhoff FM, Glass N, Holtzman D (2012) Global burden of hepatitis C: considerations for healthcare providers in the United States. Clin Infect Dis 55 Suppl 1: S10–15. 22715208 10.1093/cid/cis361

[2] Allen SA, Spaulding AC, Osei AM, Taylor LE, Cabral AM, et al (2003) Treatment of chronic hepatitis C in a state correctional facility. Ann Intern Med 138: 187–190. 12558357 10.7326/0003-4819-138-3-200302040-00010

[3] Ward JW, Rein DB, Smith BD (2012) Data to guide the “test and treat era”. of hepatitis C. Gastroenterology 143: 887–889. 22922026 10.1053/j.gastro.2012.08.027

[4] Vickerman P, Martin N, Hickman M (2011) Can Hepatitis C virus treatment be used as a prevention strategy? Additional model projections for Australia and elsewhere. Drug Alcohol Depend 113: 83–85. 20832198 10.1016/j.drugalcdep.2010.08.001

[5] DOHA (2012) Communicable Diseases Intelligence, Australia's notifiable diseases status, 2010: Annual report of the National Notifiable Diseases Surveillance System - Results: Summary, and Table 3 to 8. Australian Government, Department of Health and Ageing Available: http://www.health.gov.au/internet/main/publishing.nsf/content/cda-cdi3601a4.htm. Accessed 16 April 2013.

[6] The Kirby Institute (2012) HIV, viral hepatitis and sexually transmissible infections in Australia Annual Surveillance Report 2012. The Kirby Institute, the University of New South Wales, Sydney, NSW 2052.

[7] Butler T, Lim D, Callander D (2011) National Prison Entrants' Bloodborne Virus and Risk Behaviour Survey Report 2004, 2007, and 2010. Kirby Institute (University of New South Wales) and National Drug Research Institute (Curtin University).

[8] Walsh N, Lim M, Hellard M (2008) Using a surveillance system to identify and treat newly acquired hepatitis C infection. J Gastroenterol Hepatol 23: 1891–1894. 19120877 10.1111/j.1440-1746.2008.05508.x

[9] Gidding HF, Topp L, Middleton M, Robinson K, Hellard M, et al (2009) The epidemiology of hepatitis C in Australia: notifications, treatment uptake and liver transplantations, 1997–2006. J Gastroenterol Hepatol 24: 1648–1654. 19798783 10.1111/j.1440-1746.2009.05910.x

[10] Butler T, Milner L (2003) The 2001 Inmate Health Survey. Sydney: NSW Corrections Health Service. ISBN: 0 7347 3560 X.

[11] Butler T, Boonwaat L, Hailstone S, Falconer T, Lems P, et al (2007) The 2004 Australian prison entrants' blood-borne virus and risk behaviour survey. Australian & New Zealand Journal of Public Health 31: 44–50. 17333608 10.1111/j.1753-6405.2007.00009.x

[12] Ghany MG, Nelson DR, Strader DB, Thomas DL, Seeff LB, et al (2011) An update on treatment of genotype 1 chronic hepatitis C virus infection: 2011 practice guideline by the American Association for the Study of Liver Diseases. Hepatology 54: 1433–1444. 21898493 10.1002/hep.24641 PMC3229841

[13] Dai CY (2010) Treatment uptake of patients with chronic hepatitis C: can we expect and do more? Dig Dis Sci 55: 3300–3303. 20976622 10.1007/s10620-010-1452-6

[14] Grebely J, Genoway KA, Raffa JD, Dhadwal G, Rajan T, et al (2008) Barriers associated with the treatment of hepatitis C virus infection among illicit drug users. Drug Alcohol Depend 93: 141–147. 17997050 10.1016/j.drugalcdep.2007.09.008

[15] Grebely J, Oser M, Taylor LE, Dore GJ (2013) Breaking down the barriers to hepatitis C virus (HCV) treatment among individuals with HCV/HIV coinfection: action required at the system, provider, and patient levels. J Infect Dis 207 Suppl 1: S19–25. 23390301 10.1093/infdis/jis928 PMC3565594

[16] Mehta SH, Thomas DL, Sulkowski MS, Safaein M, Vlahov D, et al (2005) A framework for understanding factors that affect access and utilization of treatment for hepatitis C virus infection among HCV-mono-infected and HIV/HCV-co-infected injection drug users. AIDS 19 Suppl 3: S179–189. 16251816 10.1097/01.aids.0000192088.72055.90

[17] Treloar C, Newland J, Rance J, Hopwood M (2010) Uptake and delivery of hepatitis C treatment in opiate substitution treatment: perceptions of clients and health professionals. J Viral Hepat 17: 839–844. 20070504 10.1111/j.1365-2893.2009.01250.x

[18] Wagner GJ, Ryan GW (2005) Hepatitis C virus treatment decision-making in the context of HIV co-infection: the role of medical, behavioral and mental health factors in assessing treatment readiness. AIDS 19 Suppl 3: S190–198. 16251817 10.1097/01.aids.0000192089.54130.b6

[19] Osilla KC, Ryan G, Bhatti L, Goetz M, Witt M, et al (2009) Factors that influence an HIV coinfected patient's decision to start hepatitis C treatment. AIDS Patient Care STDS 23: 993–999. 19929229 10.1089/apc.2009.0153 PMC2832645

[20] Scheft H, Fontenette DC (2005) Psychiatric barriers to readiness for treatment for hepatitis C Virus (HCV) infection among injection drug users: clinical experience of an addiction psychiatrist in the HIV-HCV coinfection clinic of a public health hospital. Clin Infect Dis 40 Suppl 5: S292–296. 15768337 10.1086/427443

[21] Treloar C, Holt M (2008) Drug treatment clients' readiness for hepatitis C treatment: implications for expanding treatment services in drug and alcohol settings. Aust Health Rev 32: 570–576. 18666886 10.1071/ah080570

[22] Prochaska J, DiClemente C, Norcorss J (1992) In search of how people change: Applications to addictive behaviors. American Psychologist 47: 1102–1114. 1329589 10.1037//0003-066x.47.9.1102

[23] Holt D, Armenakis A, Harris S, Hubert S (2007) Toward a comprehensive definition of readiness for change: A review of research and instrumentation. Research in Organizational Change and Development 16: 289–336.

[24] Anderson RM (1995) Revisiting the behavioral model and access to medical care: Does it matter? J Health Soc Behav 36: 1–10. 7738325

[25] Boonwaat L, Haber PS, Levy MH, Lloyd AR (2010) Establishment of a successful assessment and treatment service for Australian prison inmates with chronic hepatitis C. Med J Aust 192: 496–500. 20438418 10.5694/j.1326-5377.2010.tb03605.x

[26] Khaw FM, Stobbart L, Murtagh MJ (2007) ‘I just keep thinking I haven't got it because I'm not yellow’: a qualitative study of the factors that influence the uptake of Hepatitis C testing by prisoners. BMC Public Health 7: 98. 17555573 10.1186/1471-2458-7-98 PMC1906754

[27] Maru DS, Bruce RD, Basu S, Altice FL (2008) Clinical outcomes of hepatitis C treatment in a prison setting: feasibility and effectiveness for challenging treatment populations. Clin Infect Dis 47: 952–961. 18715156 10.1086/591707 PMC4847716

[28] Post J, Arain A, Lloyd A (2013) Enhancing assessment and treatment of hepatitis C in the custodial setting. Clin Infect Dis 57: S70–S74. 23884069 10.1093/cid/cit265

[29] Lloyd AR, Clegg J, Lange J, Stevenson A, Post JJ, et al (2013) Safety and effectiveness of a nurse-led outreach program for assessment and treatment of chronic hepatitis C in the custodial setting. Clin Infect Dis 56: 1078–1084. 23362288 10.1093/cid/cis1202

[30] Glaser B, Strauss A (1967) The Discovery of Grounded Theory: Strategies for Qualitative Research. Chicago, IL: Aldine Publishing Co.

[31] Coupland H, Day C, Levy MT, Maher L (2009) Promoting equitable access to hepatitis C treatment for Indo-Chinese injecting drug users. Health Promot J Austr 20: 234–240. 19951245 10.1071/he09234

[32] Grebely J, Tyndall MW (2011) Management of HCV and HIV infections among people who inject drugs. Curr Opin HIV AIDS 6: 501–507. 22001894 10.1097/COH.0b013e32834bcb36

[33] Strathdee SA, Latka M, Campbell J, O'Driscoll PT, Golub ET, et al (2005) Factors associated with interest in initiating treatment for hepatitis C Virus (HCV) infection among young HCV-infected injection drug users. Clin Infect Dis 40 Suppl 5: S304–312. 15768339 10.1086/427445 PMC2196220

[34] McNally S, Latham R (2009) Recognising and Responding to Hepatitis C in Indigenous Communities in Victoria: A research project exploring barriers to hepatitis C treatment. The Australian Research Centre in Sex, Health and Society. La Trobe University.

[35] McLaren M, Garber G, Cooper C (2008) Barriers to hepatitis C virus treatment in a Canadian HIV-hepatitis C virus coinfection tertiary care clinic. Can J Gastroenterol 22: 133–137. 18299730 10.1155/2008/949582 PMC2659131

[36] Norman J, Walsh NM, Mugavin J, Stoove MA, Kelsall J, et al (2008) The acceptability and feasibility of peer worker support role in community based HCV treatment for injecting drug users. Harm Reduct J 5: 8. 18298862 10.1186/1477-7517-5-8 PMC2291043

[37] Crofts N, Louie R, Loff B (1997) The Next Plague: Stigmatization and Discrimination Related to Hepatitis C Virus Infection in Australia. Health Hum Rights 2: 86–97. 10381830

[38] Bacon BR, Gordon SC, Lawitz E, Marcellin P, Vierling JM, et al (2011) Boceprevir for previously treated chronic HCV genotype 1 infection. N Engl J Med 364: 1207–1217. 21449784 10.1056/NEJMoa1009482 PMC3153125

[39] Zeuzem S, Andreone P, Pol S, Lawitz E, Diago M, et al (2011) Telaprevir for retreatment of HCV infection. N Engl J Med 364: 2417–2428. 21696308 10.1056/NEJMoa1013086

[40] Dore GJ (2012) The changing therapeutic landscape for hepatitis C. Med J Aust 196: 629–632. 22676877 10.5694/mja11.11531

