# Supplementary material for: A Descriptive Model of Patient Readiness, Motivators, and Hepatitis C Treatment Uptake among Australian Prisoners
Source: PLoS One. 2014 Feb 27;9(2):e87564. doi: 10.1371/journal.pone.0087564 (PMC3937313; doi:10.1371/journal.pone.0087564)
Supplement: Table S1 — HePATO Qualitative Research Interview Question Guidelines. (DOCX) [file pone.0087564.s001.docx]

**Supplemental table legends:**

**Table S1:**

**Hepatitis C, Prisons, and Treatment Opportunities Study (HePATO): Qualitative Research Interview Question Guidelines (Prisoners in Qld NSW WA)**

**Background**

Can you tell me a little bit about yourself and how you came to prison? (Probe: socio-demographics, criminal history, lifestyle etc.)

**Hepatitis C**

Can you tell me about the first time you heard of hepatitis C? (Probe: place and situation)

Before you were diagnosed, what did you know about hepatitis C? (Probe: etiologies, symptoms, conventional/alternative perceptions and treatments, post-treatments)

After diagnosis, what did you know about it? (Probe)

Where did you go to for information? (Probe)

**Hepatitis C transmission**

How did you come to be infected? (Probe: drug use and sharing injecting equipment, needle stick injury, blood transfusion, blood spills in prison, fights in prison)

**Diagnosis**

Can you tell me about the time when you first diagnosed? (Probe: transmission, blood test outside of prison and on entry, who disclosed results, how he/she felt when they found out their diagnosis, last test results, and current hepatitis C status)

Is there any way we can improve your diagnosis experience inside prison?

**Hepatitis C status**

Do you know your current HCV status? What does that mean? (Probe)

Do you know your current viremic status? What does that mean? (Probe: HCV+, PCR+, cleared with treatment, auto-remitted)

Do you know what HCV genotypes you were diagnosed? What does that mean? (Probe)

**Pre and post-test counselling**

Can you recall what your health care provider told you before you were tested for hepatitis C? (Probe)

And after you were diagnosed with hepatitis C, can you recall what your health provider told you? (Probe)

**HCV decisions on treatment**

Where did you first hear of hepatitis C treatment? (Probe responses)

Before you were diagnosed, what did you know about hepatitis C treatment? And after diagnosis? (Probe responses)

Can you tell me why you decided to commence or not commence treatment? (Probe responses)

**Hepatitis C treatment**

Can you tell me the details of your treatment? What happened? (Probe: consultations, pre-treatment workups, side effects, comorbidities, discontinuation etc.)

Can you tell me about your experiences undergoing hepatitis C treatment? (Probe: assessment process, dosing, obvious benefits, side effects)

Is there any way we can improve hepatitis C treatment inside prison? (Probe)

**Support**

Did you have any support while you were on treatment? (Probe: family support, clinical support, prisoner support, emotional supports, etc.)

**Reinfection**

What are your chances of being re-infected with hepatitis C after treatment inside prison? Why? (Probe)

How about outside prison? (Probe)

**Other**

Is there anything else you would like to add on taking up hepatitis C treatment or improving your diagnosis/treatment experience inside prison?
